# The Effects of Chicory Aqueous Extract on Antioxidant Capacity, Immune Response, and Intestinal Function in Broilers

**DOI:** 10.3390/ani16193063

**Published:** 2026-09-29

**Authors:** Mingyu Wei, Mengfei Li, Zehao Li, Hao Liu, Yongzhan Bao, Ruixi Zhang, Wanyu Shi, Dou Dou, Xiao Wang

**Affiliations:** 1 College of Traditional Chinese Veterinary Medicine, Hebei Agricultural University, Baoding 071000, China; 15033257417@163.com (M.W.); limengfei0501@163.com (M.L.); 17333451727@163.com (Z.L.); 18732694087@163.com (H.L.); baoyongzhan2025@hebau.edu.cn (Y.B.); shiwanyu@hebau.edu.cn (W.S.); 2 National Animal Disease Monitoring Point of the Ministry of Agriculture and Rural Affairs, Baoding 071000, China; 3 School of Humanities, Beijing University of Chinese Medicine, Beijing 100029, China; zhangruixi16888@163.com; 4 Hebei Provincial Veterinary Biotechnology Innovation Center, Baoding 071000, China; 5 College of Life Sciences, Hebei Agricultural University, Baoding 071000, China; 6 Hebei Provincial Key Laboratory of Traditional Chinese Veterinary Medicine, Baoding 071000, China

**Keywords:** chicory aqueous extract, broiler, antioxidant capacity, immune response, intestinal function

## Abstract

In the broiler production industry, antibiotics such as florfenicol are widely used. Although these antibiotics exert therapeutic effects against bacterial diseases, they can induce a series of physiological alterations in broilers under certain exposure scenarios. This study aimed to investigate the effects of chicory aqueous extract (CAE) on broiler growth performance, immune function, and intestinal health, and to compare the impacts of CAE and florfenicol exposure on the physiological status of broilers. A total of 200 one-day-old broilers were randomly divided into five groups (5 replicates per group): the blank control group (NC) drank tap water freely, the florfenicol group (FFC) was given 0.15 g/L of florfenicol solution in the drinking water, the CAE groups were given 1.25 g/L (LCAE), 2.5 g/L (MCAE), and 5 g/L (HCAE) of CAE in the drinking water, respectively. The experimental period was 42 days. The results showed that the addition of CAE to drinking water could significantly improve the growth performance of broilers, specifically manifested as increased body weight and a significantly decreased feed-to-gain ratio (F/G) (*p* < 0.05). Regarding immune function, CAE enhanced immune capacity by increasing immune organ indices, serum antibody titers, and serum levels of immunoglobulin A (IgA) and immunoglobulin G (IgG) (*p* < 0.05). In terms of intestinal function, CAE significantly increased villus height (VH) and villus-crypt ratio (V/C), elevated activities of digestive enzymes including chymotrypsin, α-amylase, and lipase, and raised both secretory immunoglobulin A (sIgA) content and Occludin mRNA relative expression levels (*p* < 0.05), thereby improving intestinal digestion and absorption. Additionally, CAE enhanced serum superoxide dismutase (SOD) activity and reduced malondialdehyde (MDA) content (*p* < 0.05), effectively enhancing the antioxidant capacity of broilers. Notably, compared with the NC group and all CAE-treated groups, broilers in the FFC group exhibited significantly reduced growth performance, suppressed immune function, and evident damage to intestinal tissue structure and physiological functions. Comprehensive analysis indicates that CAE can effectively promote broiler growth and development, enhance immune status and intestinal digestive-absorptive function, and enrich gut bacteria such as *Lactobacillus* and *Bifidobacterium*, thus optimizing intestinal microbiota composition. Among the gradient doses tested in this study, the 2.5 g/L dose of CAE demonstrated the best overall effect, suggesting that CAE may exert beneficial physiological effects under the present experimental conditions, and further investigations are needed to assess its practical application potential.

## Simple Summary

Sustainable broiler production is essential to secure adequate supplies of high-quality animal protein. However, long-term administration of florfenicol in broiler production leads to retarded growth, immunosuppression, intestinal damage, and bacterial resistance. The objective of this study was to compare the effects of chicory aqueous extract (CAE) and florfenicol exposure on the physiological status of broilers. The trial results showed that CAE exerted dose-dependent beneficial effects, with efficacy increasing as the supplemental dose increased. It significantly improved broilers’ growth efficiency, immune status, and antioxidant capacity, optimized jejunal morphology, increased digestive enzyme activity, reinforced intestinal mucosal barriers, enriched microbiota including *Lactobacillus* and *Bifidobacterium*, and further optimized intestinal microbiota composition. In comparison, florfenicol caused comprehensive impairment of broilers’ physiological functions, and the supplementation dose of 2.5 g/L achieved the optimal comprehensive effect. This study shows that CAE shows promise as a phytogenic intervention for improving broiler health and reducing unnecessary antimicrobial use in poultry production.

## 1. Introduction

Chicken is an important source of high-quality animal protein for humans [1]. To support the sustainable development of broiler farming, CAE may be further evaluated as a phytogenic intervention to support broiler health and reduce unnecessary antimicrobial use. However, in the large-scale broiler farming system, antibiotic products have long been used to prevent and treat bacterial infections in poultry, effectively reducing the incidence of bacterial diseases and thereby improving farming efficiency and economic benefits [2]. Florfenicol (FFC) is a monofluorinated derivative of chloramphenicol [3], and its mechanism of action is similar to that of chloramphenicol. It can tightly bind to the 50S subunit of the bacterial 70S ribosome, inhibiting protein synthesis and thereby hindering bacterial proliferation [4]. Owing to the rapid absorption, strong tissue penetration, mild side effects, low cost, and potent inhibitory activity of florfenicol against both Gram-positive and Gram-negative bacteria, florfenicol ranks first among veterinary antibiotics applied in Chinese broiler farming, with a farm usage rate of 78% [5]. Although countries such as China have banned the addition of antibiotics to animal feed, drugs like florfenicol are still permitted as therapeutic veterinary medicines for the prevention and treatment of bacterial diseases in livestock and poultry. In broiler production practice, florfenicol is commonly administered during the chick rearing phase to regulate intestinal microbiota and ensure the early health of chicks [6].

However, with the long-term and widespread use of florfenicol, its application effects and potential risks have increasingly attracted attention. Studies have shown that oral administration of florfenicol can lead to changes in the intestinal structure, imbalance of the microbial community, and cause adverse effects such as growth and development disorders, abnormal energy metabolism, and damage to tissues and organs [7]. Moreover, the long-term use of florfenicol can also induce bacteria to develop resistance and accelerate the spread of resistance genes [8]. The emergence of resistant strains will significantly weaken the antibacterial effect of florfenicol, leading to an increase in the infection probability of broilers. Resistance genes not only increase the difficulty of treating diseases and raise the incidence rate and mortality rate, but also significantly increase the production costs. Such risks may further intensify with the extension of the medication period [9]. Currently, the abuse of antibiotics has become a global public health issue. In response to the potential threat posed by bacterial resistance to human health and production activities, gradually reducing the use of antibiotics in livestock and poultry production and discovering and applying new drugs have become inevitable trends for the high-quality development of the future livestock industry.

Relevant studies have shown that the application of plant extracts as natural and green additives in poultry farming can effectively enhance production performance and improve product quality [10]. Chicory, also known as bitter chicory, French chicory, and blue chicory, is a perennial herb with both medicinal and edible value. The main plant compounds it contains include inulin, coumarin, tannins, flavonoids, and sesquiterpene lactones, etc. [11,12]. Inulin, a type of indigestible carbohydrate and one of the main active components in chicory, enhances cellular immune function and promotes antibody and cytokine secretion. It is regarded as a typical prebiotic [13]. In addition to inulin, phenolic acids and flavonoids are also core bioactive compounds in chicory. Phenolic acids have been proven to possess diverse pharmacological activities, including antibacterial, antioxidant, antiviral, anti-inflammatory, anti-ulcer, and anti-tumor effects [14]. Flavonoids are natural polyphenolic compounds widely present in fruits, vegetables, and plants. They not only exert antioxidant, anti-inflammatory, antiviral, and anti-tumor activities, but also improve insulin resistance in the body [15]. A previous study established an acute inflammatory model in mice using a 1% carrageenan saline suspension and applied chicory extract as an intervention. The results showed that the extract significantly reduced serum levels of pro-inflammatory cytokines while enhancing antioxidant capacity [16]. Currently, research on chicory primarily focuses on individual active components. However, potential interactions among different chicory constituents remain underexplored. Thus, studies on the native multi-component system of chicory and its aqueous extract in broiler production are still limited. Therefore, this study focused on CAE rich in natural complex components, systematically investigating its effects on broiler growth performance, immune function, and intestinal health. As widely recognized, the intestine serves not only as a central organ for nutrient metabolism and immune regulation but also functions as the first line of defense against external stressors, toxins, and pathogens via its epithelial tissues [17,18,19]. Hence, exploring the role of CAE in maintaining intestinal mucosal integrity and physiological stability holds significant scientific importance for ensuring proper nutrient digestion, absorption, and overall health in poultry.

Therefore, this study aimed to evaluate the biological effects of chicory aqueous extract and compare the impacts of CAE and florfenicol exposure on the physiological status of broilers. Meanwhile, a positive control group treated with florfenicol via drinking water was established [20,21,22]. To analyze the differential responses of growth, immunity, antioxidant capacity, intestinal status, and cecal microbiota in broilers under the two treatments, and provide experimental evidence for understanding the physiological activity of chicory aqueous extract (CAE) and organismal effects induced by florfenicol exposure.

## 2. Materials and Methods

### 2.1. Preparation of CAE

The chicory slices were purchased from Ronghua Bencao Traditional Chinese Medicine Materials Co., Ltd. (Anguo City, Baoding, Hebei Province, China). The preparation method of the CAE was as follows: chicory slices were pre-soaked in deionized water for 30 min, then were extracted with 10-fold and 8-fold volumes of deionized water at 80 °C for 1 h, respectively, followed by filtration. The filtrate was collected and evaporated using a rotary evaporator (RE-501, Nanbei Instrument Co., Ltd., Zhengzhou, China), with the speed set at 60 r. A circulating water-type multi-purpose vacuum pump (SHB-III, Zhengzhou Greatwall Scientific Industrial and Trade Co., Ltd., Zhengzhou, Henan, China) was used for vacuum water circulation. A low-temperature cooling circulator (DLK-2020, Ningbo Scientz Biotechnology Co., Ltd., Ningbo, Zhejiang, China) was used for water cooling, with the temperature set at 4 °C. The final extract was adjusted to a concentration of 1 g raw material per 1 mL solution.

### 2.2. Analysis and Identification of CAE Components

In total, 100 μL of sample was taken and mixed with 400 μL of extraction solution (MeOH:ACN, 1:1 (*v*/*v*)). The extraction solution contains deuterated internal standards; the mixed solution was vortexed for 30 s, sonicated for 10 min in a 4 °C water bath, and incubated for 1 h at −40 °C to precipitate proteins. Transfer 200 μL of liquid to the well of a 0.22 μm filter plate. Place the plate on the manifold. Apply vacuum, 4 psi, 90 s. Take the plate from the positive pressure device for analysis. For non-polar metabolites, LC-MS/MS analyses were performed using a UHPLC system (Vanquish, Thermo Fisher Scientific, Waltham, MA, USA) with a Phenomenex Kinetex C18 (2.1 mm × 50 mm, 2.6 μm) coupled to an Orbitrap Exploris 120 mass spectrometer (Orbitrap MS, Thermo). The mobile phase A:0.01% acetic acid in water; mobile phase B:IPA:ACN (1:1, *v*/*v*). Column temperature: 25 °C. The autosampler temperature was 4 °C, and the injection volume was 2 μL. The Orbitrap Exploris 120 mass spectrometer was used for its ability to acquire MS/MS spectra in information-dependent acquisition (IDA) mode under the control of the acquisition software (Xcalibur (v4.4), Thermo). In this mode, the acquisition software continuously evaluates the full scan MS spectrum. The ESI source conditions were set as follows: sheath gas flow rate as 50 Arb, Aux gas flow rate as 15 Arb, capillary temperature 320 °C, Sweep Gas: 1 Arb, Vaporizer Temp: 350 °C, full MS resolution as 60,000, MS/MS resolution as 15,000, collision energy: SNCE 20/30/40, spray voltage as 3.8 kV (positive) or −3.4 kV (negative), respectively. The raw data were converted to the mzXML format using ProteoWizard and processed with an in-house program, which was developed using R and based on XCMS, for peak detection, extraction, alignment, and integration. The R package and the BiotreeDB (V3.0, Shanghai Biotree Biomedical Technology Co., Ltd., Shanghai, China) were applied in metabolite identification.

### 2.3. Experimental Design and Animal Husbandry Management

In this experiment, 200 one-day-old, healthy, and nearly equal-weight Arbor Acres (AA) broilers were obtained from Xingrui Agriculture and Husbandry Development Co., Ltd., Baoding, China. They were randomly and evenly divided into five groups, with five replicates per group and eight broilers per replicate. According to the preliminary experimental design, the blank control group (NC) had free access to tap water, and the florfenicol group (FFC) received 0.15 g/L of florfenicol solution in their drinking water, which was purchased from Zhonglong Shenli Animal Pharmaceutical Co., Ltd. in Hefei, China. The low (LCAE), medium (MCAE), and high (HCAE) groups were supplemented with 1.25, 2.5, and 5 mL of concentrated CAE into 1 L of tap water, respectively, resulting in final concentrations of 1.25, 2.5, and 5 g/L in the diluted drinking water. Each batch was prepared in 3 L volumes and distributed into three separate water dispensers for free access by broiler chickens, with the water changed twice daily, morning and evening. The concentration gradient used in this study was based on previously reported dosages of similar plant extracts in broilers, with upper and lower limits determined through preliminary trials. A doubling progression was adopted to clearly reveal dose–response relationships and facilitate linear and quadratic regression analyses, thereby providing a basis for identifying the optimal addition level.

Medication was administered from day 1, continuously for 5 days, with a 7-day interval, followed by another 5 days of medication. Broilers were euthanized, and relevant samples were collected at 21 and 42 days of age, respectively. The experimental period lasted for 42 days. At 7 days of age, broilers were immunized subcutaneously at the neck with a combined inactivated vaccine against Newcastle disease and avian influenza (H9N2 subtype) at a dosage of 0.3 mL per bird. At 21 days of age, broilers were immunized intranasally or ocularly with a live Newcastle disease vaccine at a dosage of 0.05 mL per bird. The experiment was conducted during summer at the Animal Laboratory of Hebei Agricultural University. Warm white LED lighting with a color temperature of 2700–3000 K was used for photoperiod control. The stocking density was maintained at 10 birds per m^2^, and relative humidity was kept between 55% and 60%. The lighting regimen was as follows: during the first week, light intensity was maintained at 30–40 lux, then gradually reduced to 5–10 lux thereafter. The initial photoperiod was set at 23 h of light and 1 h of darkness, which was adjusted to 18 h of light and 6 h of darkness before day 35. Temperature management was implemented as follows: ventilation and cooling equipment were installed in the barn to maintain an ambient temperature of 32 ± 3 °C during the first week, decreasing by 2 °C each subsequent week until reaching a stable temperature of 26 °C from the fourth week onward, maintained throughout the remainder of the trial. The experiment employed open-sided pens, each equipped with two feed troughs and three water dispensers. Daily observations and recordings of residual water volume in each group’s water dispensers showed no significant differences among groups, indicating consistent intake of CAE across all treatment groups. Feed was provided ad libitum at fixed times each day. The basic feed was formulated according to the “Chicken Feeding Standards” (NY/T 33-2004) [23]. The composition and nutritional levels were shown in Table 1. The metabolizable energy was calculated according to the “Chinese Feed Composition and Nutritional Value Table (33rd Edition, 2022)” [24].

### 2.4. Growth Performance

During the feeding period, residual feed was weighed and discarded daily. Daily feed intake and broiler body weight were recorded. At 21 and 42 days of age, eight broilers per group were randomly selected for weighing and sample collection, and average daily gain (ADG), average daily feed intake (ADFI), and feed-to-gain ratio (F/G) were calculated for these two growth stages. ADG = (final body weight − initial body weight)/number of days in the experiment, ADFI = total feed consumption/(experimental days × number of broilers), F/G = ADFI/ADG.

### 2.5. Immune Organ Indices

At 21 and 42 days of age, eight broilers per group were randomly selected for weighing and then euthanized. The entire thymus, spleen, and bursa of Fabricius were isolated, and any surrounding excess tissue was trimmed away. The surface blood was blotted dry with filter paper, and the organs were weighed to calculate the immune organ indices. The immune organ index was defined as the ratio of the organ weight to the corresponding body weight (mg/g).

### 2.6. Blood Collection and Serum Analyses

#### 2.6.1. Blood Collection and Serum Preparation

At 21 and 42 days of age, blood samples were collected from the wing vein of eight broilers per group for immunoglobulin and antioxidant assays. For ND and AI antibody monitoring, blood samples were collected at weekly intervals throughout the experimental period (on immunization days, birds were immunized at 6:00 a.m. and blood was collected after 6:00 p.m.). Serum was separated by centrifugation at 3000× *g* for 15 min at 4 °C and stored at −20 °C until further analysis.

#### 2.6.2. Measurement of Serum Immunoglobulin Contents

Serum concentrations of IgA, IgG, and immunoglobulin M (IgM) were assessed using commercial ELISA kits (Yuanju Biotechnology Center, Shanghai, China; Cat. No. 202506). The absorbance was measured at 450 nm using a microplate reader (Multiskan Ascent, Thermo Fisher Scientific Inc., Waltham, MA, USA). All procedures were strictly conducted in accordance with the manufacturer’s instructions.

#### 2.6.3. Measurement of Serum ND Antibody and AI (H9N2 Subtype) Antibody Levels

Hemagglutination inhibition (HI) assay was used to assess serum antibody titers against Newcastle disease (ND) virus and avian influenza (AI) virus (H9N2 subtype). Briefly, serum samples were serially diluted (2-fold) in phosphate-buffered saline (PBS, pH 7.2) and mixed with an equal volume of 4 HAU of ND or AI (H9N2) antigen. After incubation at 37 °C for 30 min, an equal volume of 1% chicken red blood cell suspension was added, and the mixture was incubated at room temperature for 30 min. Antibody titers were expressed as log_2_ of the reciprocal of the highest serum dilution showing complete hemagglutination inhibition. Geometric mean titers (GMTs) were calculated for each group at each sampling time point.

#### 2.6.4. Measurement of Serum Antioxidant Capacity Levels

Serum levels of malondialdehyde (MDA; Cat. No. A003-1-2), total superoxide dismutase (T-SOD; Cat. No. A001-3-2) activity, and reduced glutathione (GSH; Cat. No. A006-2-1) were measured using commercial assay kits (Nanjing Jiancheng Bioengineering Institute, Nanjing, China). The absorbance was read at 532 nm, 450 nm, and 405 nm, respectively, using a microplate reader (Multiskan Ascent, Thermo Fisher Scientific Inc., Waltham, MA, USA). All procedures were strictly conducted in accordance with the manufacturer’s protocols.

### 2.7. Measurement of Jejunal Mucosal Morphology

At 21 and 42 days of age, six broilers per group were randomly selected for weighing and then euthanized; the abdominal cavity was opened, and a segment of approximately 2 cm long jejunum was excised at the same position and preserved in formalin solution. The fixed tissue samples underwent processes including rinsing, dehydration, clearing, wax infiltration, embedding, and hematoxylin–eosin (H&E) staining. Under an optical microscope, the structural changes in the intestinal tissue of the broilers were observed, and the VH and Crypt Depth (CD) were measured. The V/C was calculated as V/C = VH/CD.

### 2.8. Measurement of Jejunal Digestive Enzyme Activity

At 21 and 42 days of age, eight broilers per group were randomly selected for weighing and then euthanized. Add 100 mg of jejunal chyme to 900 μL of pre-cooled saline solution, homogenize mechanically, and centrifuge at 3000 r for 10 min at 4 °C. Collect the supernatant for parameter analysis. Protein concentration in jejunal chyme was assessed using a reagent kit (Beyotime Biotechnology Co., Ltd., Shanghai, China). Chymotrypsin activity in jejunal chyme was assayed using a reagent kit (AidiSheng Biotechnology Co., Ltd., Yancheng, Jiangsu, China). Additionally, α-amylase and lipase activities in jejunal chyme were assayed using a reagent kit (Nanjing Jiancheng Biological Engineering Institute, Nanjing, China). All procedures were strictly conducted in accordance with the manufacturer’s instructions.

### 2.9. Measurement of Mucosal Barrier-Related Factor Expression in the Jejunum

At 21 and 42 days of age, eight broilers per group were randomly selected for weighing and then euthanized. Add 100 mg of jejunal tissue to 900 μL of pre-cooled saline solution, homogenize mechanically, and centrifuge at 3000 r for 10 min at 4 °C. Collect the supernatant for parameter analysis. The content of sIgA in the jejunum was assessed using an ELISA kit (Shanghai Yuanju Biotechnology Center, Shanghai, China). All procedures were strictly conducted in accordance with the manufacturer’s instructions.

Total RNA was extracted with the Eastep^®^ Super Total RNA Extraction Kit (Promega, Shanghai, China), and the purity and concentration of RNA were measured using the NanoOne Ultra Micro Spectrophotometer (Yooning Instrument Co., Ltd., Hangzhou, China). Then, it was reverse transcribed into cDNA using the All-In-One 5X RT MasterMix Kit (Applied Biological Materials, Vancouver, BC, Canada). Primers listed in Table 2 were used, and the sample was added strictly following the instructions provided by the BlasTaqTM 2X qPCR MasterMix Kit (Applied Biological Materials, Vancouver, BC, Canada). After all operations were completed, real-time quantitative polymerase chain reaction (RT-qPCR) analysis was performed using a fluorescence quantitative PCR system (Bioer, Hangzhou, China). The RT-qPCR cycling conditions were as follows: enzyme activation at 95 °C for 3 min, denaturation at 95 °C for 15 s, and annealing at 60 °C for 1 min, for a total of 40 cycles. GAPDH was used as the internal reference gene, and the relative mRNA expression levels of the relevant genes were calculated using the 2^−ΔΔCt^ method. The mRNA expression levels of mucosal barrier-related factors including Occludin, Claudin-1, and ZO-1 were assessed.

### 2.10. Immunohistochemical Analysis of Mucosal Barrier-Related Factors in the Jejunum

At 21 and 42 days of age, six broilers per group were randomly selected, and 2 cm-long jejunal segments were collected and fixed in 4% neutral buffered formalin, embedded in paraffin, and sectioned at 4 μm. Following baking at 60 °C for 2 h, sections were deparaffinized using xylene substitute (G1128, Wuhan Servicebio Technology Co., Ltd., Wuhan, China; 3 × 10 min), rehydrated in a graded ethanol series, and rinsed with distilled water. Antigen retrieval was carried out in 1 × Tris-EDTA buffer (pH 8.0, diluted 1:20 from 20 × stock solution G1206, Servicebio) at 95–100 °C for 30 min. Slides were then allowed to cool naturally (20–30 min) and washed three times with PBS (G0002, Servicebio) for 5 min per wash. Endogenous peroxidase activity was blocked with 3% H_2_O_2_ for 25 min at room temperature in the dark, and non-specific antigen-binding sites were blocked with 3% BSA (GC305010, Servicebio) for 30 min.

Sections were incubated overnight (12–16 h) at 4 °C with primary antibodies diluted 1:500 in PBS: rabbit anti-Occludin (GB111401, Servicebio), rabbit anti-Claudin-1 (GB152543, Servicebio), and rabbit anti-ZO-1 (GB151981, Servicebio). After washing, sections were incubated with ready-to-use S-vision polymer goat anti-rabbit secondary antibody (G1302, Servicebio) for 50 min at room temperature. DAB chromogenic reagent (G1212, Servicebio; A:B = 50:1) was applied, and chromogenic development was monitored under a light microscope for 1–5 min. Cell nuclei were counterstained with hematoxylin (G1004, Servicebio) for 3 min, differentiated (G1039) for 2–3 s, and blued (G1040) for 30 s. Subsequently, sections were dehydrated, cleared, and mounted with neutral balsam (10004160, Sinopharm Chemical Reagent Co., Ltd., Shanghai, China). Positive and negative controls (PBS used in place of the primary antibody) were included for each staining batch. Images were acquired using a digital slide scanner.

### 2.11. Cecal Microbiome Analysis

At 42 days of age, six broilers per group were randomly selected, and cecal contents were collected for genomic DNA extraction. Genomic DNA was extracted from the collected cecal contents using the fecal genomic DNA extraction kit (DP328, TIANGEN BIOTECH (Beijing, China) Co., Ltd.). After assessing DNA purity and concentration, the samples were diluted to 1 ng/μL with sterile water. Using the diluted genomic DNA as the template, the full-length 16S rRNA gene was amplified by PCR. The primer sequences were as follows: Forward: AGRGTTYGATYMTGGCTCAG; Reverse: RGYTACCTTGTTACGACTT. Subsequently, the PCR products were pooled in equal amounts and purified according to their concentrations to construct SMRTbell libraries, which were then sequenced on the PacBio sequencing platform. Raw sequencing data were demultiplexed according to barcode sequences using the Lima (v2.9.0) software, and all sample sequences were stored in BAM format. Sequence correction was performed using CCS (SMRT Link v7.0) with CCS = 3 and a minimum accuracy of 0.99. Sequences with lengths shorter than 1340 bp or longer than 1740 bp were filtered out, and the qualified sequences were saved in FASTQ and FASTA formats. Subsequently, SSR filtering and primer trimming were performed using Cutadapt (v3.7). Sequences containing more than eight consecutive identical bases were removed. The final reads obtained after the above quality control procedures were defined as valid clean reads.

All clean reads were clustered into operational taxonomic units (OTUs) using the Uparse (v7.0.1001) software, and sequences were clustered into OTUs by default at 97% identity. The most frequently occurring sequence in each OTU was selected as the representative sequence for subsequent taxonomic annotation. Taxonomic classification of representative OTU sequences was performed using the Mothur algorithm based on the SILVA 138.1 SSU rRNA database, with a confidence threshold set from 0.8 to 1.0.

Data analysis and visualization were performed using Uparse (v7.0.1001), QIIME (v1.9.1), R (v2.15.3) and LEfSe (v1.1.2) software. To characterize the intestinal microbiota, α-diversity was analyzed using the ACE, Chao1, Simpson, and Shannon indices. *β*-diversity was evaluated by PCoA, and the relative abundance of microbiota was calculated at the phylum and genus levels. Finally, LEfSe analysis was used to identify differentially abundant microbial communities.

### 2.12. Statistical Analysis

Data organization and statistical calculations were performed using Microsoft Excel 2021. Normality and homogeneity of variance were tested prior to analysis. Statistical analyses were conducted using SPSS 26.0 software. Group comparisons were carried out using one-way analysis of variance (ANOVA). If significant differences were found, Tukey’s multiple comparison test was applied, followed by linear and quadratic regression analyses. The FFC group was excluded from the linear and quadratic regression analyses. Data are presented as mean ± SEM. All graphs were generated using GraphPad Prism 10. *p* < 0.05 was considered statistically significant, *p* < 0.01 indicated highly significant differences, and *p* > 0.05 indicated no statistically significant difference.

## 3. Results

### 3.1. Results of Analysis and Identification of CAE Components

**Figure 1:**
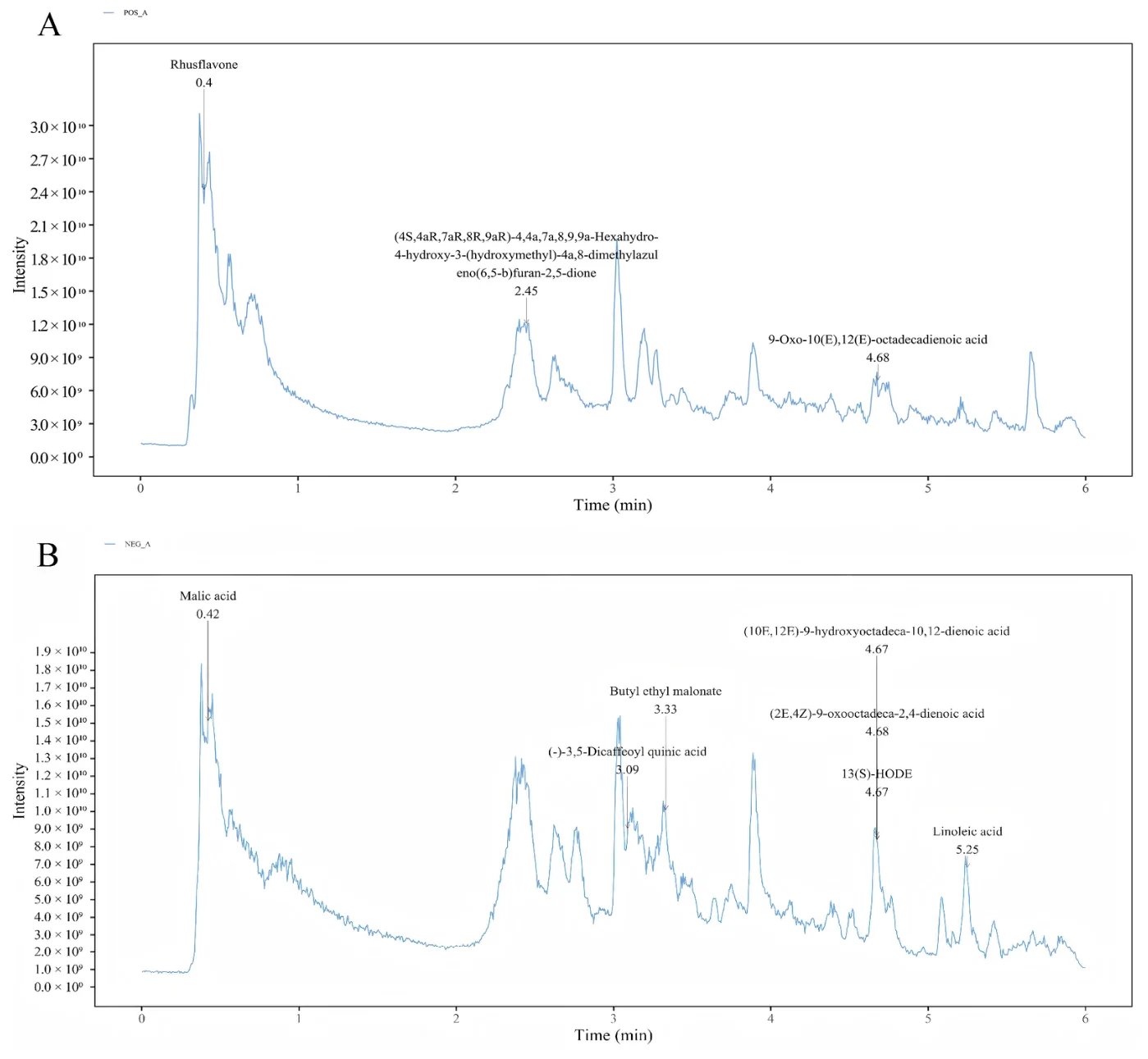
Total ion chromatograms of CAE in both positive and negative ion modes. (**A**) Positive ion mode of CAE; (**B**) negative ion mode of CAE.

Among the measured components, 490 types matched the standard substances at Level 1, 438 types matched the public library at Level 2, 134 types matched the theoretical library at Level 3, and 56,533 types were unknown compounds at Level 4. The ten components with the highest relative contents are as follows: (4S,4aR,7aR,8R,9aR)-4,4a,7a,8,9,9a-Hexahydro-4-hydroxy-3-(hydroxymethyl)-4a,8-dimethylazuleno(6,5-b)furan-2,5-dione, Rhusflavone, 13(S)-HODE, (10E,12E)-9-hydroxyoctadeca-10,12-dienoic acid, Malic acid, (−)-3,5-Dicaffeoyl quinic acid, (2E,4Z)-9-oxooctadeca-2,4-dienoic acid, 9-Oxo-10(E),12(E)-octadecadienoic acid, Butyl ethyl malonate and Linoleic acid (Table 3). As shown in Figure 1, the total ion chromatograms of CAE were obtained under positive and negative ion modes.

### 3.2. Effects of CAE on the Growth Performance of Broilers

As shown in Table 4, during the 1–21 days of age, compared with the NC group, the ADG of the FFC group was significantly decreased (*p* < 0.05), and the F/G was significantly increased (*p* < 0.05). There were no significant differences in ADG and F/G among the CAE groups (*p* > 0.05). However, both indicators showed significant linear and quadratic dose effects with increasing supplementation level of CAE (*p* < 0.05). Compared with the FFC group, the ADG of the CAE groups was significantly increased (*p* < 0.05), and the F/G was significantly decreased (*p* < 0.05). During 22–42 days of age, the one-way ANOVA showed no significant differences in F/G among treatment groups (*p* > 0.05), while F/G showed significant linear and quadratic dose effects (*p* < 0.05). Compared with the FFC group, the ADG of the LCAE and MCAE groups was significantly increased (*p* < 0.05). During the 1–42 days of age, compared with the NC group, the ADG of the FFC group was significantly decreased (*p* < 0.05), while the ADG of the MCAE group was significantly increased (*p* < 0.05), and ADG showed a significant quadratic dose effect with increasing supplementation level of CAE (*p* < 0.05). The F/G of the MCAE and HCAE groups was significantly decreased (*p* < 0.05) and showed significant linear and quadratic dose effects (*p* < 0.05), reaching the lowest value in the MCAE group. Compared with the FFC group, the ADG of the CAE groups was significantly increased (*p* < 0.05), and the F/G was significantly decreased (*p* < 0.05).

### 3.3. Effects of CAE on the Immune Organ Indices of Broilers

As shown in Table 5, at 21 days of age, compared with the NC group, the bursa of Fabricius index of the FFC group was significantly decreased (*p* < 0.05), and the spleen and bursa of Fabricius index in the MCAE and HCAE groups were significantly increased (*p* < 0.05). There was no significant difference in the thymus index among the LCAE, MCAE, and HCAE groups (*p* > 0.05). All the above indicators exhibited significant linear and quadratic dose effects with increasing supplementation level of CAE (*p* < 0.05). Compared with the FFC group, the spleen index of the MCAE and HCAE groups, as well as the thymus and bursa of Fabricius indices of the LCAE, MCAE, and HCAE groups were significantly increased (*p* < 0.05). At 42 days of age, compared with the NC group, no significant differences were observed in the spleen and thymus index among the LCAE, MCAE, and HCAE groups (*p* > 0.05), while the bursa of Fabricius index in the HCAE group was significantly increased (*p* < 0.05). The thymus index and bursa of Fabricius index showed significant linear and quadratic dose effects with increasing supplementation level of CAE (*p* < 0.05). Compared with the FFC group, the thymus and bursa of Fabricius indices of the MCAE and HCAE groups were significantly increased (*p* < 0.05).

### 3.4. Effects of CAE on Serum Immunoglobulin Contents in Broilers

**Figure 2:**
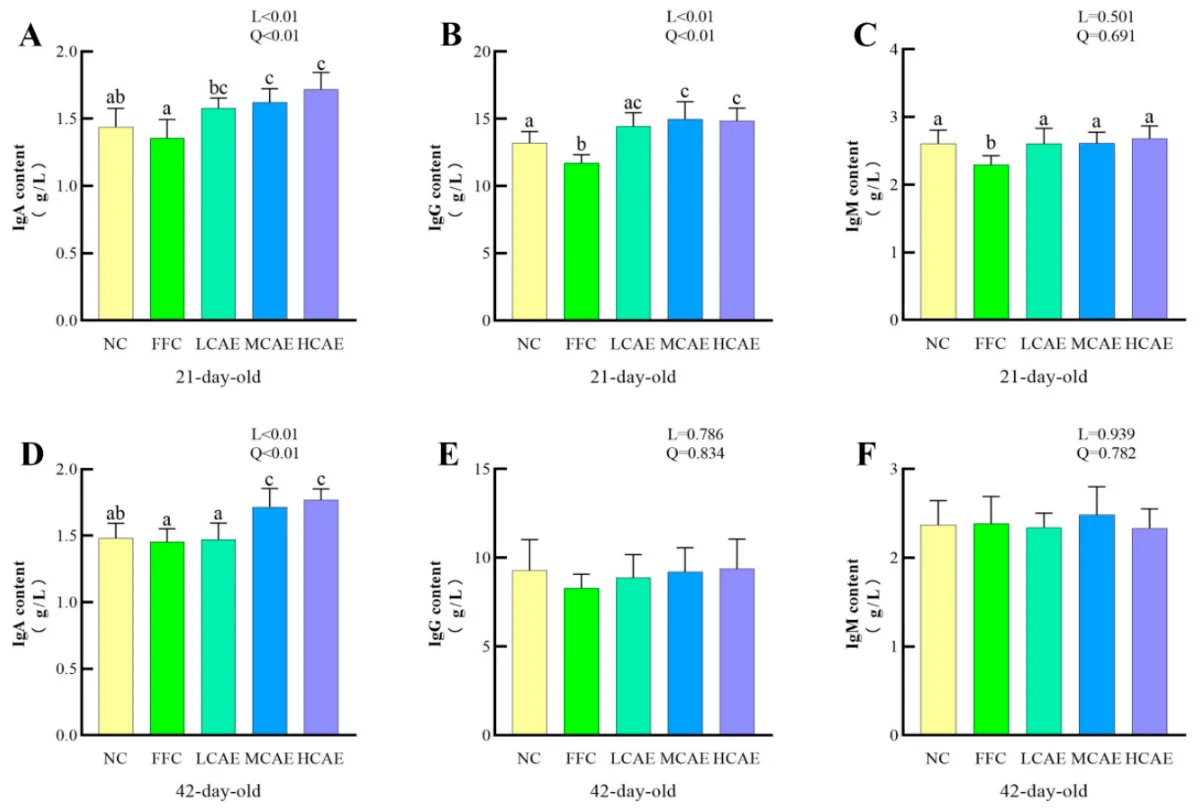
Effects of CAE on serum immunoglobulin contents in broilers. (**A**–**C**) The effect on the contents of IgA, IgG, and IgM in the serum at 21 days of age. (**D**–**F**) The effect on the contents of IgA, IgG, and IgM in the serum at 42 days of age. Different superscript letters indicate significant differences (*p* < 0.05). The NC group was allowed to freely drink tap water; the FFC group was given 0.15 g/L florfenicol solution in drinking water; the LCAE, MCAE, and HCAE groups were respectively given 1.25, 2.5, and 5 g/L CAE in drinking water. SEM, standard error of the mean; A, *p*-value of one-way ANOVA; L, *p*-value of linear analysis; Q, *p*-value of quadratic analysis. *n* = 8.

As shown in Figure 2, at 21 days of age, compared with the NC group, the contents of IgG and IgM in the FFC group were significantly decreased (*p* < 0.05), while the contents of IgA and IgG in the MCAE and HCAE groups were significantly increased (*p* < 0.05). Moreover, both the contents of IgA and IgG showed significant linear and quadratic dose effects with increasing supplementation level of CAE (*p* < 0.05). Compared with the FFC group, the contents of IgA, IgG, and IgM in the LCAE, MCAE, and HCAE groups were significantly increased (*p* < 0.05). At 42 days of age, compared with the NC and FFC groups, the content of IgA in the MCAE and HCAE groups was significantly increased (*p* < 0.05), and IgA showed significant linear and quadratic dose effects with increasing supplementation level of CAE (*p* < 0.05). There were no significant differences in the contents of IgG and IgM among the dose groups (*p* > 0.05).

### 3.5. Effects of CAE on the Changes in Serum ND Antibody and AI (H9N2 Subtype) Antibody Levels in Broilers

**Figure 3:**
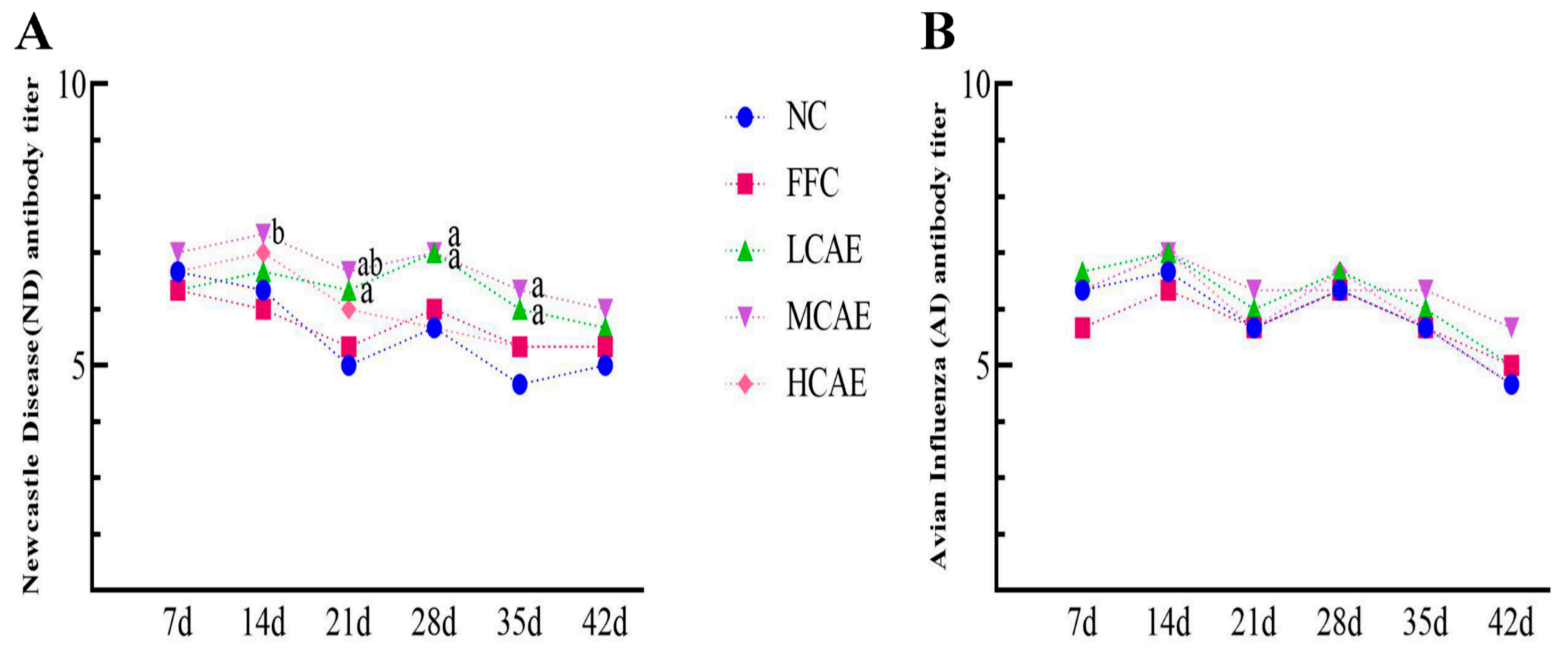
Effect of CAE on antibody titers in the serum of broilers. (**A**) Newcastle disease antibody titer. (**B**) Avian influenza antibody titer. a indicates a significant difference compared with the NC group (*p* < 0.05). b indicates a significant difference compared with the FFC group (*p* < 0.05). *n* = 8.

As shown in Figure 3, at 14 days of age, compared with the FFC group, the Newcastle disease (ND) antibody titer in the MCAE group was significantly increased (*p* < 0.05). At 21 days of age, compared with the NC group, the ND antibody titer in the LCAE group was significantly increased (*p* < 0.05). Compared with both the NC and FFC groups, the ND antibody titer in the MCAE group was significantly increased (*p* < 0.05). At 28 and 35 days of age, compared with the NC group, ND antibody titers in the LCAE and MCAE groups were significantly increased (*p* < 0.05). There were no significant differences in avian influenza (AI) antibody titers among the various dose groups (*p* > 0.05).

### 3.6. Effects of CAE on Serum Antioxidant Capacity Levels in Broilers

**Figure 4:**
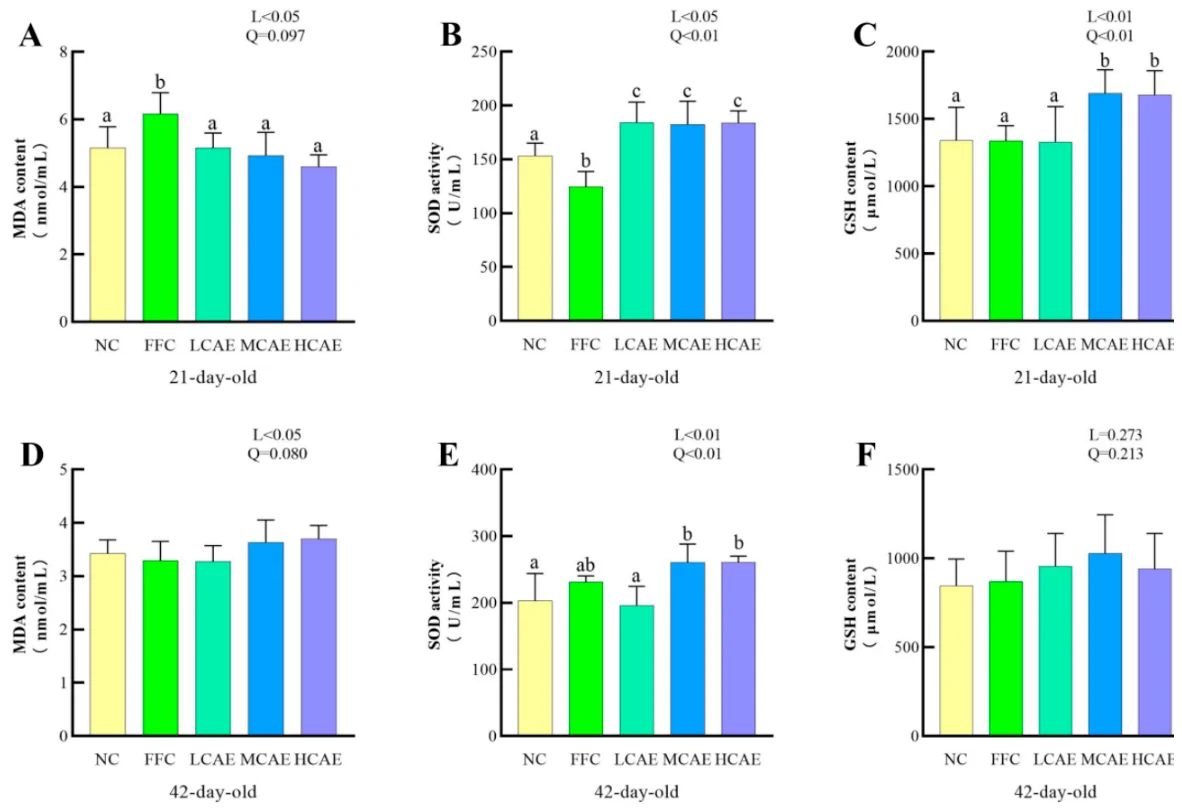
Effect of CAE on serum antioxidant capacity levels in broilers. (**A**–**C**) The effect on the levels of MDA, SOD, and GSH in the serum at 21 days of age. (**D**–**F**) The effect on the levels of MDA, SOD, and GSH in the serum at 42 days of age. Different superscript letters indicate significant differences (*p* < 0.05). *n* = 8.

As shown in Figure 4, at 21 days of age, compared with the NC group, the level of MDA in the FFC group was significantly increased (*p* < 0.05), and the level of SOD was significantly decreased (*p* < 0.05). Compared with the NC and FFC groups, the level of SOD in the LCAE, MCAE, and HCAE groups and the level of GSH in the MCAE and HCAE groups were significantly increased (*p* < 0.05), and both the levels of SOD and GSH showed significant linear and quadratic dose effects with increasing supplementation level of CAE (*p* < 0.05). At 42 days of age, compared with the NC group, the level of SOD in the MCAE and HCAE groups was significantly increased (*p* < 0.05), and the level of SOD showed significant linear and quadratic dose effects with increasing supplementation level of CAE (*p* < 0.05). There were no significant differences in the levels of MDA and GSH among all dose groups (*p* > 0.05).

### 3.7. Effects of CAE on the Jejunal Histomorphology of Broilers

**Figure 5:**
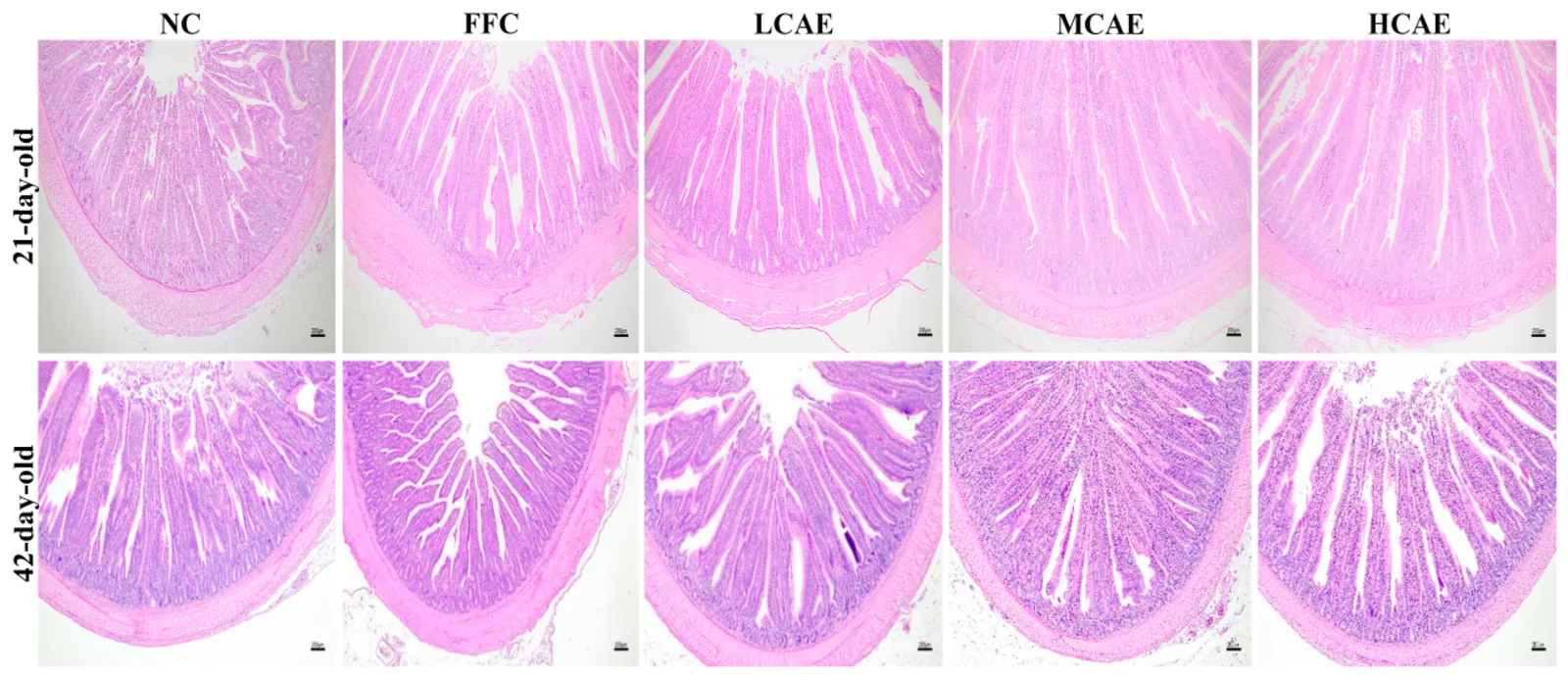
Jejunal morphological structure of broilers in each group under light microscope (HE staining, 40×). *n* = 6.

As shown in Figure 5, at 21 days of age, the intestinal villi in the NC group were intact and neatly arranged. The villi in the FFC group were sparse and relatively loose in structure. All CAE groups showed intact villus structure with no obvious pathological damage, and the villi in the MCAE group were arranged relatively tightly. At 42 days of age, the villi in the NC group were regular and intact in morphology. The villi in the FFC group were disordered and showed obvious breakage. The villi in the LCAE and HCAE groups were intact and regular in structure, and those in the MCAE group were tightly arranged with a complete and orderly structure.

As shown in Table 6, at 21 days of age, compared with the NC group, the jejunal VH and V/C in the FFC group were significantly decreased (*p* < 0.05), while the jejunal V/C in the MCAE and HCAE groups was significantly increased (*p* < 0.05). Compared with the FFC group, the jejunal VH and V/C in the LCAE, MCAE, and HCAE groups were significantly increased (*p* < 0.05). At 42 days of age, compared with the NC and FFC groups, the jejunal VH in the LCAE, MCAE, and HCAE groups was significantly increased (*p* < 0.05), and the jejunal V/C in the MCAE and HCAE groups was significantly increased (*p* < 0.05). At both 21 and 42 days of age, the jejunal VH and V/C showed significant linear and quadratic dose effects with increasing supplementation level of CAE (*p* < 0.05).

### 3.8. Effects of CAE on Digestive Enzyme Activity in the Jejunum of Broilers

**Figure 6:**
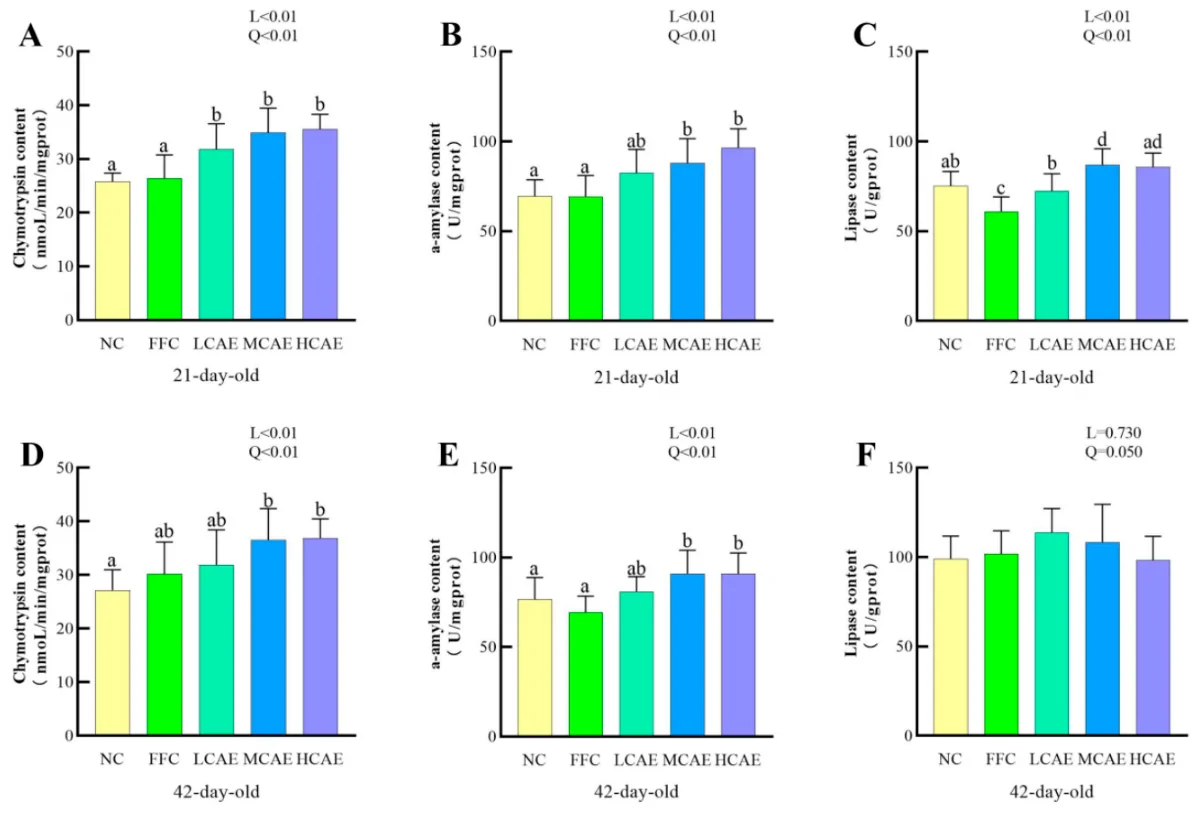
Effects of CAE on digestive enzyme activities in the jejunum of broilers. (**A**–**C**) Effects on chymotrypsin activity, α-amylase activity, and lipase activity in the jejunum at 21 days of age. (**D**–**F**) Effects on chymotrypsin activity, α-amylase activity, and lipase activity in the jejunum at 42 days of age. Different superscript letters indicate significant differences (*p* < 0.05). *n* = 8.

As shown in Figure 6, at 21 days of age, compared with the NC and FFC groups, the jejunal chymotrypsin activity in the LCAE, MCAE, and HCAE groups and the jejunal α-amylase activity in the MCAE and HCAE groups were significantly increased (*p* < 0.05). Compared with the NC group, the jejunal lipase activity in the FFC group was significantly decreased (*p* < 0.05), while the jejunal lipase activity in the MCAE group was significantly increased (*p* < 0.05). Compared with the FFC group, the jejunal lipase activity in the LCAE, MCAE, and HCAE groups was significantly increased (*p* < 0.05). All the above indicators showed significant linear and quadratic dose effects with increasing supplementation level of CAE (*p* < 0.05). At 42 days of age, compared with the NC group, the jejunal chymotrypsin activity in the MCAE and HCAE groups was significantly increased (*p* < 0.05). Compared with the NC and FFC groups, the jejunal α-amylase activity in the MCAE and HCAE groups was significantly increased (*p* < 0.05). There was no significant difference in jejunal lipase activity among all dose groups (*p* > 0.05). Meanwhile, both jejunal chymotrypsin and α-amylase activities showed significant linear and quadratic dose effects with increasing supplementation level of CAE (*p* < 0.05).

### 3.9. Effects of CAE on Jejunal Mucosal Barrier-Related Factor Expression in Broilers

**Figure 7:**
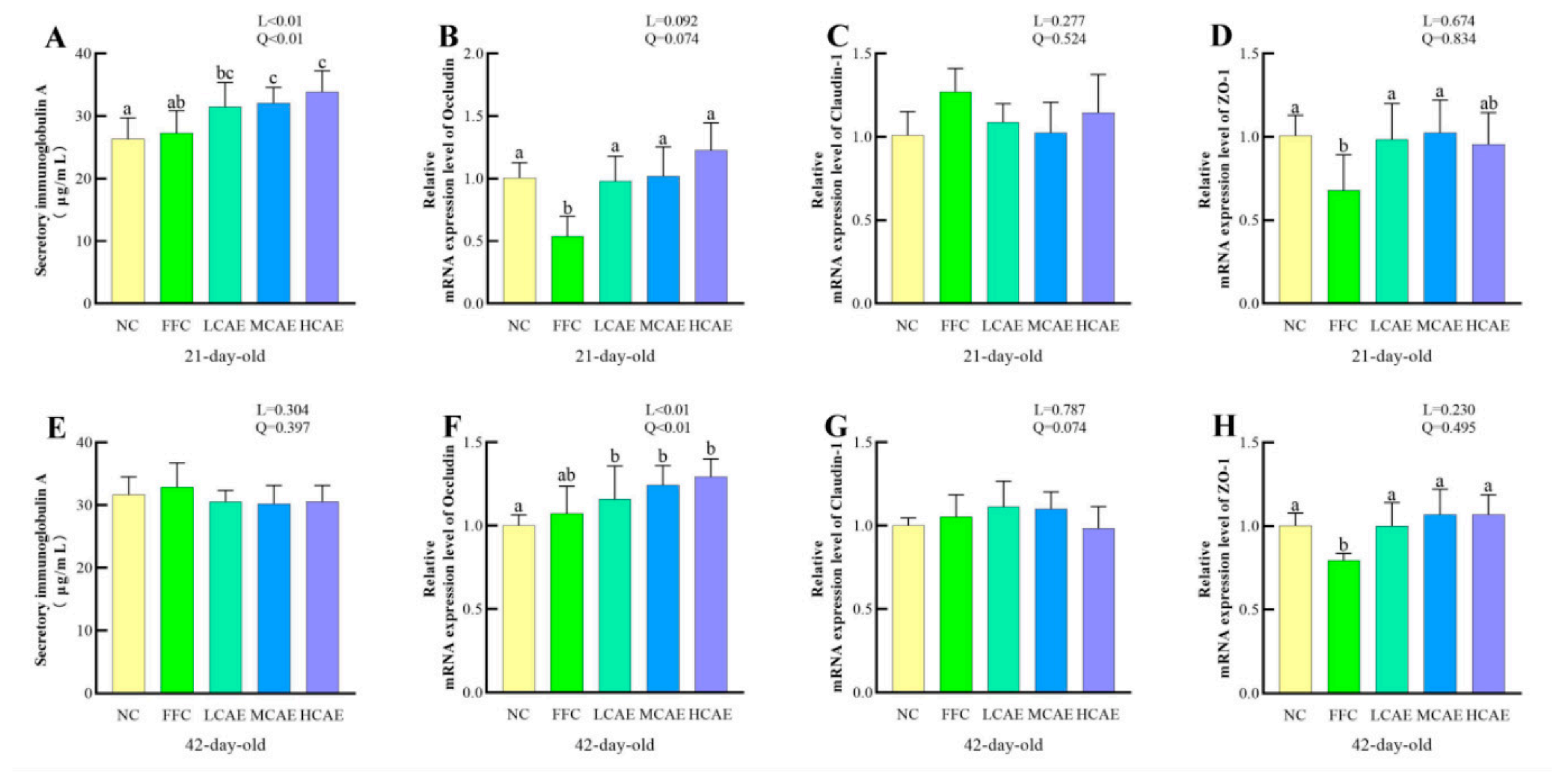
Effects of CAE on factors related to the jejunal mucosal barrier in broilers. (**A**–**D**) Effects on the contents of sIgA, Occludin, Claudin-1 and ZO-1 in the jejunum at 21 days of age. (**E**–**H**) Effects on the contents of sIgA, Occludin, Claudin-1 and ZO-1 in the jejunum at 42 days of age. Different superscript letters indicate significant differences (*p* < 0.05). *n* = 8.

As shown in Figure 7, at 21 days of age, compared with the NC group, the content of jejunal sIgA in the LCAE, MCAE, and HCAE groups was significantly increased (*p* < 0.05). Compared with the FFC group, the content of jejunal sIgA in the MCAE and HCAE groups was significantly increased (*p* < 0.05). However, the jejunal sIgA showed significant linear and quadratic dose effects with increasing supplementation level of CAE (*p* < 0.05). At 42 days of age, there was no significant difference in the content of jejunal sIgA among the LCAE, MCAE, and HCAE groups (*p* > 0.05). Additionally, at 21 days of age, compared with the NC group, the mRNA expression of jejunal Occludin and ZO-1 in the FFC group was significantly decreased (*p* < 0.05). Compared with the FFC group, the mRNA expression of jejunal Occludin in the LCAE, MCAE, and HCAE groups was significantly increased (*p* < 0.05), and the mRNA expression of jejunal ZO-1 in the MCAE and HCAE groups was significantly increased (*p* < 0.05). At 42 days of age, compared with the NC group, the mRNA expression of jejunal Occludin in the LCAE, MCAE, and HCAE groups was significantly increased (*p* < 0.05), and the mRNA expression of jejunal ZO-1 in the FFC group was significantly decreased (*p* < 0.05). Compared with the FFC group, the mRNA expression of jejunal ZO-1 in the LCAE, MCAE, and HCAE groups was significantly increased (*p* < 0.05). However, the mRNA expression of jejunal Occludin showed significant linear and quadratic dose effects with increasing supplementation level of CAE (*p* < 0.05). There were no significant differences in the mRNA expression of jejunal Claudin-1 among the various dose groups (*p* > 0.05).

### 3.10. Effect of CAE on the Expression and Distribution of Jejunal Mucosal Barrier-Related Factors in Broilers

**Figure 8:**
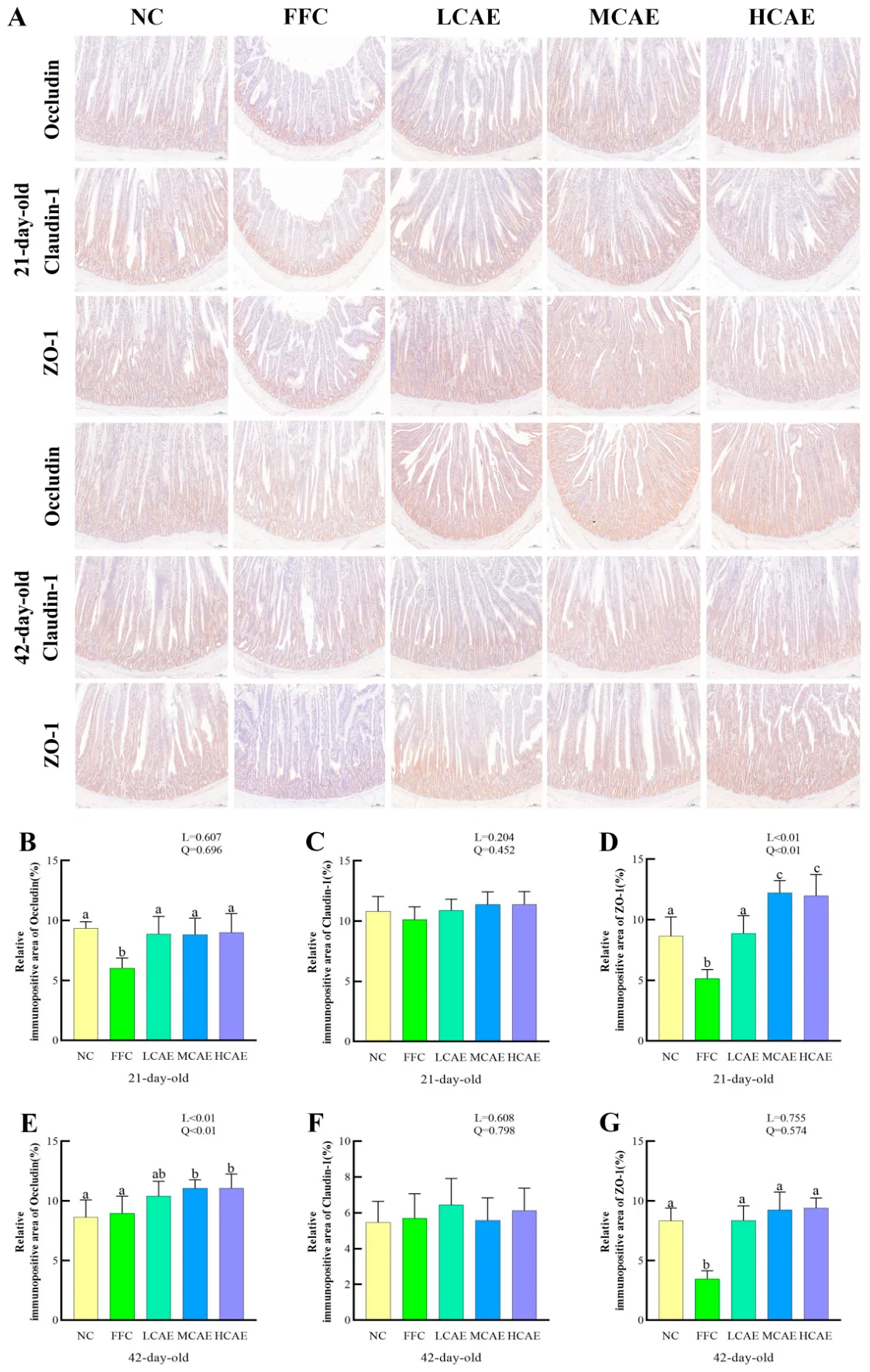
Effect of CAE on jejunal mucosal barrier by immunohistochemistry in broilers. (**A**) The effect on intestinal mucosal barrier immunity at 21 and 42 days of age. (**B**–**D**) The immunopositive expression of Occludin, Claudin-1 and ZO-1 in the intestine of 21-day-old broilers. (**E**–**G**) The immunopositive expression of Occludin, Claudin-1 and ZO-1 in the intestine of 42-day-old broilers. Different superscript letters indicate significant differences (*p* < 0.05) (IHC staining, 40×). *n* = 6.

As shown in Figure 8, at 21 days of age, compared with the NC group, the immunopositivity of jejunal Occludin and ZO-1 in the FFC group was significantly decreased (*p* < 0.05). Compared with the NC and FFC groups, the immunopositivity of jejunal ZO-1 in the MCAE and HCAE groups was significantly increased (*p* < 0.05). However, the immunopositivity level of ZO-1 in the jejunum showed significant linear and quadratic dose effects with increasing supplementation level of CAE (*p* < 0.05). At 42 days of age, compared with the NC and FFC groups, the immunopositivity of jejunal Occludin in the MCAE and HCAE groups was significantly increased (*p* < 0.05). Additionally, compared with the NC group, the immunopositivity of jejunal ZO-1 in the FFC group was significantly decreased (*p* < 0.05). However, the immunopositivity level of Occludin in the jejunum showed significant linear and quadratic dose effects with increasing supplementation level of CAE (*p* < 0.05). There were no significant differences in the immunopositivity of Claudin-1 in the jejunum among the various dose groups (*p* > 0.05).

### 3.11. Effect of CAE on the Enumeration of Operational Taxonomic Units (OTUs) in Cecal Contents

**Figure 9:**
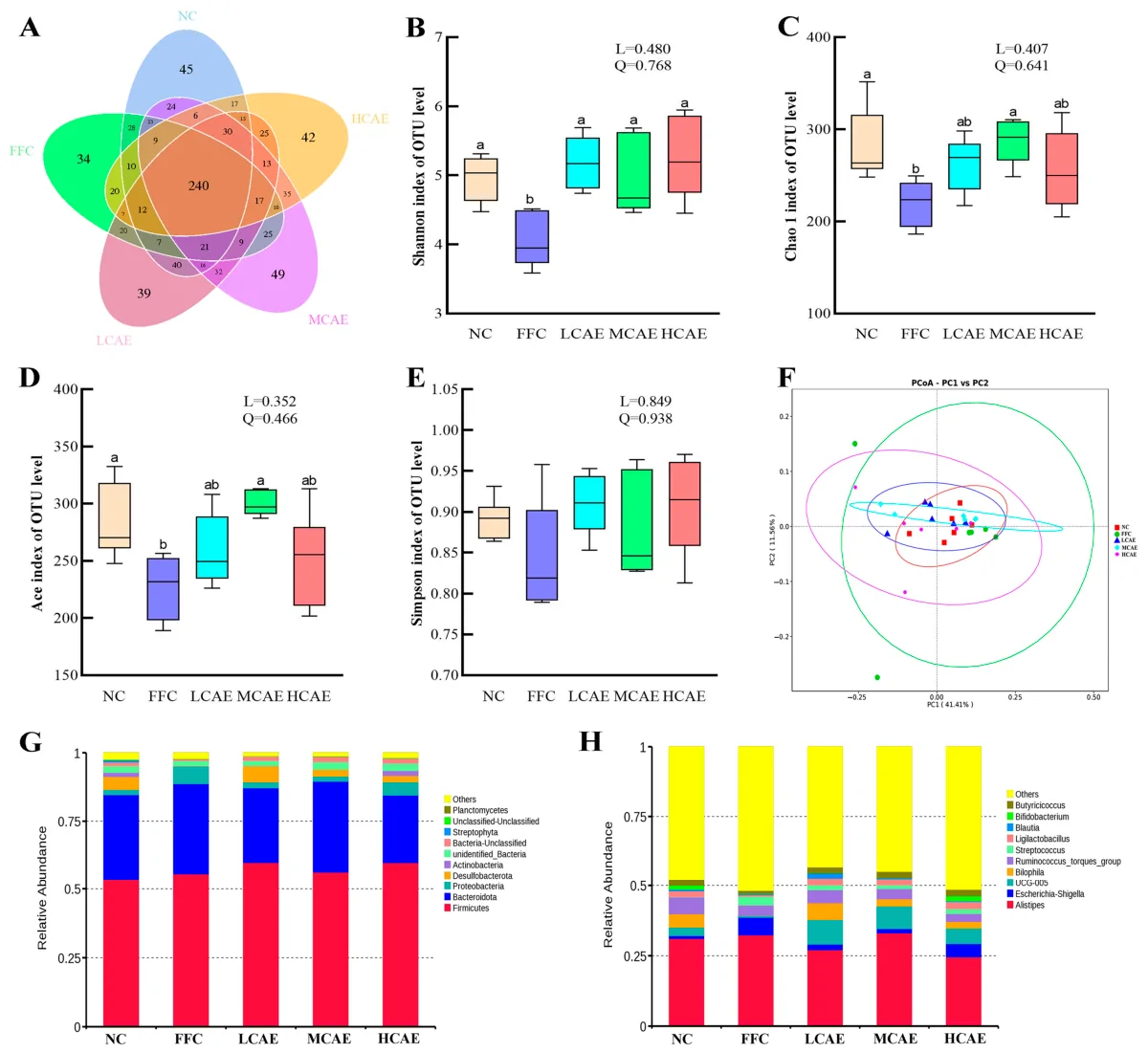
Analysis of effective operational taxonomic units, microbial diversity, and relative abundance of cecal microflora at the phylum and genus levels in broilers. (**A**) Effective operational taxonomic units of cecal contents. (**B**–**E**) α-diversity of cecal contents. (**F**) *β*-diversity of cecal contents. (**G**) Microbial community distribution at the phylum level (stacked bar chart). (**H**) Microbial community distribution at the genus level (stacked bar chart). Different superscript letters indicate significant differences (*p* < 0.05). *n* = 6.

Figure 9A revealed that the NC group contained 543 OTUs, the FFC group contained 492 OTUs, the LCAE group contained 543 OTUs, the MCAE group contained 559 OTUs, and the HCAE group contained 508 OTUs. There were 240 OTUs shared among all groups. Specifically, the NC, FFC, LCAE, MCAE, and HCAE groups had 45, 34, 39, 49, and 42 unique OTUs, respectively. Notably, the number of unique OTUs in the MCAE group was higher than that in the NC and FFC groups.

### 3.12. Effect of CAE on α-Diversity and β-Diversity of Cecal Microorganisms in Broilers

Figure 9B–E revealed that, compared with the NC group, the Shannon, Chao1, and ACE indices in the FFC group were significantly decreased (*p* < 0.05). Compared with the FFC group, the Shannon index in the LCAE, MCAE, and HCAE groups was significantly increased (*p* < 0.05), and the Chao1 and ACE indices in the MCAE group were also significantly increased (*p* < 0.05). Compared with the NC group, although the Chao1 and ACE indices in the MCAE group slightly increased, the difference was not significant (*p* > 0.05). However, with increasing supplementation level of CAE, linear and quadratic dose effects of the above indicators showed no significance (*p* > 0.05). Figure 9F revealed that the PCoA analysis based on *β*-diversity showed that the contribution rates of the two principal components, PC1 and PC2, were 41.41% and 11.56%, respectively, which could effectively reflect the differential characteristics of microbial community structures among various groups. Compared with the NC group, the sample distribution within the FFC and HCAE groups was relatively scattered, indicating poor homogeneity in microbial community structures within these groups, and there were significant differences in species composition structure between these groups and the NC group. However, there were multiple overlaps in the distance of sample distribution within the NC group and the LCAE and MCAE groups.

### 3.13. Effect of CAE on the Relative Abundance of Cecal Microbiota at the Phylum and Genus Levels in Broilers

Figure 9 revealed that Panel 9G presented a stacked plot of microbial species distribution at the phylum level. The phyla with relatively high abundance were primarily *Firmicutes*, *Bacteroidota*, *Proteobacteria*, etc. Compared with the NC group (20.97%), the *Bacteroidota* relative abundance was numerically lower in the HCAE group (16.50%). Compared with the NC group (11.10%), the *Proteobacteria* relative abundance was numerically higher in the FFC group (37.54%) and HCAE group (28.29%).

Figure 9H was a stacked plot of microbial species distribution at the genus level. The 10 species with relatively high abundance mainly belonged to the genera *Alistipes*, followed by *Escherichia-Shigella*, *UCG-005*, *Bilophila*, *Ruminococcus-torques-group*, *Streptococcus*, *Ligilactobacillus*, *Blautia*, *Bifidobacterium*, and *Butyricicoccus*. Compared with the NC group (20.98%), the relative abundance of *Alistipes* was numerically lower in the LCAE group (18.29%) and HCAE group (16.58%). Compared with the NC group (6.60%), the relative abundance of *Escherichia–Shigella* was numerically higher in the FFC group (40.17%) and HCAE group (30.28%). In addition, compared with the NC group (11.58%), the relative abundance of *UCG-005* was numerically higher in the LCAE group (33.60%), MCAE group (30.92%), and HCAE group (21.29%), while the relative abundance of *UCG-005* was numerically lower in the FFC group (2.61%).

### 3.14. Effect of CAE on the LEfSe Analysis of Cecal Microbiota in Broilers

**Figure 10:**
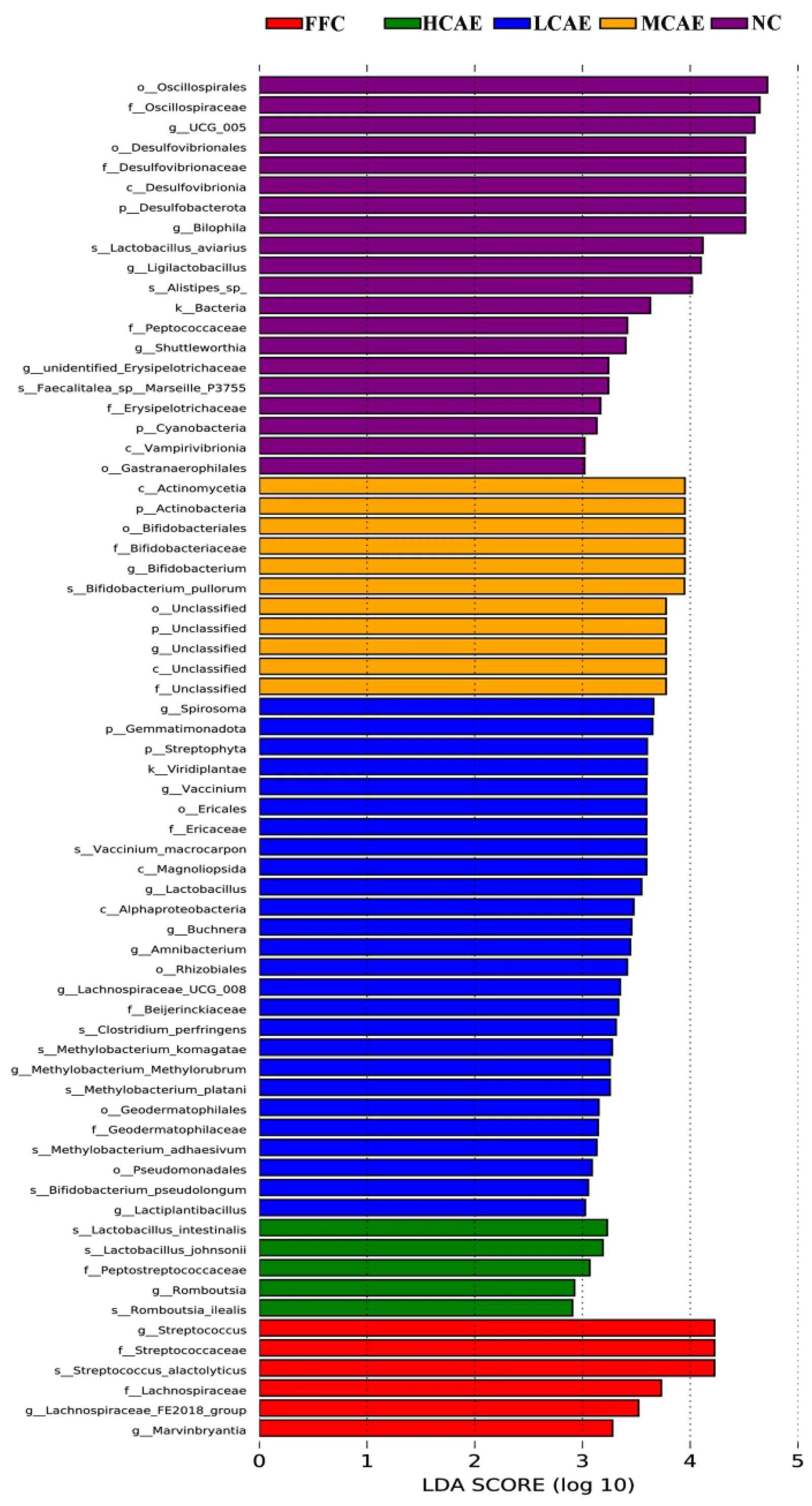
LEfSe analysis of cecal microbiota. *n* = 6.

As shown in Figure 10, when the LDA threshold was set at 2.0, differentially enriched taxa for each group were screened. Percentage values represent the mean relative abundance of corresponding taxa within each group. In the NC group, *UCG-005*, *Bilophila*, *Ligilactobacillus*, and *Shuttleworthia* were identified as differentially enriched taxa. In the FFC group, the differentially enriched taxa included *Streptococcus*, *Marvinbryantia*, and *Lachnospiraceae-FE2018-group*. In the LCAE group, the differentially enriched taxa included *Spirosoma*, *Vaccinium*, *Lactobacillus*, *Buchnera*, *Amnibacterium*, *Lachnospiraceae-UCG-008*, *Methylobacterium–Methylorubrum*, and *Lactiplantibacillus*. In the MCAE group, the differentially enriched taxa were *Bifidobacterium*. In the HCAE group, the differentially enriched taxa included *Lactobacillus* and *Romboutsia*.

## 4. Discussion

The preliminary study of this experiment completed the qualitative analysis of the chemical composition of CAE, confirming that it mainly contains active substances such as carbohydrates and glycosides, glycolipids, flavonoids, phenolic acids, sesquiterpenes, triterpenes, and ketocarboxylic acids. The results of this trial showed that the ADG in the FFC group significantly decreased, while the F/G significantly increased, consistent with previous research findings [25]. Notably, although florfenicol has good antibacterial activity, long-term application can reduce intestinal digestive enzyme activity, disrupt gut microbiota, and break intestinal microecological homeostasis, thereby causing nutrient absorption disorders and growth retardation. This was likely the reason for the lower body weight of broilers in the FFC group. Even so, florfenicol remains widely used in clinical animal production due to its strong antimicrobial properties. Considering the physiological changes induced by florfenicol exposure in broilers, this study systematically evaluated the physiological effects of graded-dose CAE intervention on broilers. Results indicated that adding different doses of CAE significantly increased ADG and decreased F/G in broilers, suggesting that CAE could improve growth performance. Notably, during the 22–42 day period, although significant linear and quadratic regression indicated a clear dose-dependent trend, F/G did not reach statistical significance.

Currently, there are few reports on the use of CAE administered via drinking water to regulate broiler growth performance. Since improved growth performance directly reflects enhanced overall health status, this study further explored the regulatory effects of CAE on immune function, intestinal morphology, intestinal digestive enzyme activity, intestinal barrier function, mucosal immunoglobulin levels, and cecal microbial community structure in broilers.

Immunity is a core physiological function of the body. The immune system distinguishes self from non-self substances, removes antigenic foreign substances via immune responses, and maintains physiological homeostasis [26]. Our findings show that both the immune organ indices and serum immunoglobulin levels were significantly decreased in the FFC group. Previous studies have confirmed that florfenicol exerts notable immunosuppressive effects, primarily by inhibiting immune cell proliferation, thus impairing host immune function [27], which explained the reduced immune status observed in the FFC group. In contrast, CAE significantly increased immune organ indices and serum IgA and IgG levels. Meanwhile, it increased Newcastle disease (ND) antibody titers to varying degrees in broilers at different growth stages, indicating that CAE effectively enhanced immune function in broilers. On the one hand, this may be attributed to the presence of active components such as phenolic compounds in chicory, which have been shown to significantly improve poultry immune status [28]. Research indicates that phenolic compounds may promote the secretion of immunoglobulins and cytokines [29], optimize intestinal physiological functions, improve nutrient absorption efficiency, and consequently enhance immune function in broilers. On the other hand, chicory polysaccharides could also possess strong immune-enhancing activities. They may indirectly activate immune-related cells and enhance phagocytic function, thereby improving the overall immune performance of broilers [30]. However, the increase in immune organ indices should not be directly regarded as evidence of enhanced immune capacity of the organism. Organ enlargement may result from either physiological immune activation or inflammatory stimulation. Combined with the experimental results of simultaneous increases in serum immunoglobulin levels and vaccine antibody titers, it collectively indicates that CAE can exert a positive regulatory effect on the immune status of broilers.

Antioxidant enzymes including SOD, catalase (CAT), and glutathione peroxidase (GSH-Px) scavenge excess reactive oxygen species and protect cells against oxidative damage [31,32]. MDA, a metabolite of lipid peroxidation, can reflect the degree of oxidative damage in the organism [33]. In the present study, the FFC group exhibited significantly increased MDA levels and decreased SOD activity. The results indicated that florfenicol aggravated oxidative stress in broilers, induced lipid peroxidation damage, and inhibited the activities of major antioxidant enzymes. This may be related to its interference with hepatic metabolic enzyme systems, leading to free radical accumulation, as well as its immunosuppressive effects, which further weakened the immature antioxidant defense mechanisms in young broilers. The SOD activity and GSH content in the CAE group were significantly increased, indicating that CAE effectively improved antioxidant enzyme levels and alleviated oxidative stress. This may be attributed to the natural antioxidant properties of phenolic compounds present in the extract.

Intestinal morphology is a fundamental basis for evaluating intestinal nutrient absorption function, with VH, CD, and V/C serving as key indicators of digestive and absorptive capacity [34,35]. Villi are the primary functional tissues responsible for nutrient absorption; higher VH correlates with stronger nutrient absorption ability. Shallower CD indicates faster maturation of intestinal epithelial cells and improved secretory function. An increased V/C typically reflects overall improvement in intestinal absorption function [36]. Previous studies have shown that dietary supplementation with chicory can increase VH and the V/C in the jejunum of poultry, thereby optimizing digestive and absorptive capacity and improving feed utilization [37]. In this study, at 21 days of age, the jejunal VH and V/C in the FFC group were significantly decreased. At 42 days of age, the CD in the FFC group was significantly decreased, suggesting that after drug withdrawal, intestinal tissue structure and physiological functions gradually recovered as the drug was metabolized. Moreover, all CAE treatment groups showed varying degrees of improvement in jejunal VH and V/C, indicating that CAE effectively optimized intestinal mucosal morphology and enhanced nutrient digestion and absorption. Additionally, inulin in chicory may inhibit the colonization of both pathogenic and non-pathogenic bacteria, reduce intestinal mucosal inflammation and damage, and thus may improve VH and CD, enhancing digestive and secretory function and nutrient absorption efficiency [38].

Intestinal digestive enzyme activity is an important indicator for assessing nutrient digestion and absorption. Chymotrypsin, amylase, and lipase are primarily secreted by small intestinal epithelial cells and the pancreas, and their activities reflect the body’s ability to utilize proteins, starch, and fat from feed [39]. We observed that lipase activity was significantly decreased in the FFC group at 21 days of age. At 42 days of age, there was no significant difference in digestive enzyme activity between the FFC and NC groups, indicating that adding florfenicol to drinking water decreased jejunal digestive enzyme activity in broilers, but intestinal function gradually recovered as birds aged following drug withdrawal. In contrast, adding CAE to drinking water significantly increased chymotrypsin, α-amylase, and lipase activities. Therefore, our findings suggest that adding CAE to drinking water increased jejunal digestive enzyme activity, thereby improving the body’s ability to digest and utilize proteins, starch, and fats. However, research on the effects of CAE on digestive enzyme activity in broilers remains limited. Nevertheless, some studies indicate that epithelial cells lining the intestinal villi are responsible for synthesizing and secreting digestive enzymes, and villus atrophy often coincides with impaired digestion and absorption, likely due to reduced enzyme production [40]. Based on this, improvements in intestinal villus morphology may underlie enhanced digestive enzyme activity. Thus, we propose that CAE may improve intestinal digestive and absorptive functions by increasing VH and the V/C ratio, thereby enhancing digestive enzyme activity.

The intestinal microbiota plays a crucial role in maintaining immune homeostasis, and microbial imbalance can lead to serious health issues such as inflammation and intestinal barrier damage [41]. sIgA is mainly synthesized by mucosal plasma cells in the gut and forms a protective layer on the intestinal epithelium, preventing pathogens and foreign antigens from invading. It also interacts with beneficial bacteria, enhancing their survival and suppressing harmful bacterial growth, thereby modulating the intestinal microbiota structure [42,43,44,45]. The results indicate that sIgA levels were significantly increased across all CAE dosage groups, indicating enhanced intestinal mucosal immunity and further supporting its regulatory effects on the gut microbiota. The intestinal physical barrier is composed of intestinal epithelial cells and intercellular junctions, with occludin, claudin, and ZO-1 being key proteins that maintain tight junctions and prevent the invasion of harmful substances [46]. In this study, the expression of the Occludin gene in the jejunum was significantly increased in the CAE group, while the expression of Occludin and ZO-1 was markedly decreased in the FFC group. The results indicated that CAE significantly enhanced the expression of tight junction proteins in the jejunum, thereby reducing intestinal permeability, improving intestinal mucosal barrier function and immune capacity, maintaining intestinal barrier integrity, and effectively enhancing broiler growth performance. In contrast, adding florfenicol to drinking water damaged the intestinal physical barrier. Additionally, research has shown that beneficial gut bacteria such as Lactobacillus can suppress harmful microbial populations, repair the intestinal barrier, and modulate microbiota composition [47]. Therefore, the integrity of the intestinal mucosal barrier is closely linked to the microbiota; increased sIgA levels and elevated expression of genes related to the intestinal mucosal barrier create favorable conditions for beneficial bacteria. At the same time, an increase in beneficial gut bacteria helps stabilize the host’s immune status, further ensuring the structural integrity of the intestinal mucosa. Notably, the elevation of beneficial bacteria induced by CAE in drinking water provides additional support for the observed increases in sIgA and intestinal mucosal barrier-related genes.

A rich and diverse microbial community promotes intestinal microbiota balance and health, helping maintain microbial homeostasis and supporting overall gut health [48]. 16S rRNA sequencing analysis revealed that the number of OTUs in the MCAE group was higher than in the NC group, indicating a greater abundance of unique taxonomic units specific to this group and suggesting a more distinctive community structure. α-diversity analysis showed that Shannon, Chao 1, and ACE indices were significantly lower in the FFC group compared to the NC group, while CAE treatment groups exhibited microbial dominance and evenness closer to those of the NC group. Overall, these findings suggested that CAE effectively regulated intestinal α-diversity, outperforming florfenicol. Furthermore, *β*-diversity analysis indicated that the microbial community structures in all chicory-treated groups were more similar to those in the NC group, whereas the FFC group showed a deviation from normal microbial profiles, demonstrating that CAE contributed to a more stable intestinal microbiota compared to florfenicol.

At the phylum level, *Firmicutes*, *Bacteroidetes* and *Proteobacteria* were identified as the dominant bacterial phyla in the gut, consistent with previous studies showing that *Firmicutes* and *Bacteroidetes* are the predominant phyla in broiler ceca [49]. Excessive proliferation of *Proteobacteria* reflected instability in the intestinal microbiota [50]. This study found no significant differences in the abundances of *Firmicutes*, *Bacteroidetes*, and *Proteobacteria* between the MCAE group and the NC group, whereas both the FFC and HCAE groups showed increased levels of *Proteobacteria*, suggesting potential microbial imbalance. At the genus level, the top three dominant genera were *Alistipes*, *Escherichia-Shigella*, and *UCG-005*. The abundance of *UCG-005* increased in both the MCAE and HCAE groups compared with the NC group, while *Escherichia-Shigella* abundance also increased in the FFC and HCAE groups. LEfSe analysis revealed that the FFC group was enriched with genera such as *Streptococcus*. In comparison, the MCAE group harbored high-abundance taxa frequently reported to be associated with favorable gut status, including *Bifidobacterium*, while the HCAE group showed a predominance of *Lactobacillus*. These findings aligned with previous research on chicory [51]. Thus, we speculated that the elevated abundances of *Bifidobacterium* and *Lactobacillus* in the MCAE and HCAE groups could be associated with the prebiotic effects of inulin. Specifically, this conclusion remains speculative and requires further experimental validation. Notably, the relative abundance of *Proteobacteria* and *Escherichia-Shigella* increased in the HCAE group. Although the high-dose group exhibited acceptable performance in other indices, these shifts serve as warning signs of potential gut microbiota dysbiosis from a microbial perspective. Nevertheless, overall experimental results indicated that different doses of CAE significantly regulated the abundance and structure of gut-associated microbial communities.

Notably, beneficial effects on intestinal health remained detectable on day 42, 25 days after the last CAE administration. This sustained response indicates that short-term intermittent CAE treatment can trigger persistent changes within the gut. We speculate that early CAE exposure may modulate intestinal microbiota and enhance the integrity of the intestinal mucosal barrier. Such improvements lay a solid foundation for intestinal development, especially villus growth, which may account for the sustained intestinal responses. The above explanations are only reasonable speculations, and further targeted studies are required to clarify the relevant physiological pathways.

This study proposed three possible mechanisms through which CAE may have improved broiler growth performance This study proposed three mechanisms through which CAE improved broiler growth performance: first, by leveraging the prebiotic effects of components such as inulin, which may promote the proliferation of beneficial bacteria and enhancing nutrient absorption, thereby supporting animal growth [52]; however, this putative mechanism has not been directly validated in the present study and requires further targeted investigation; second, through its inherent antimicrobial activity, which may modulate intestinal microbiota via competitive exclusion and improving feed conversion efficiency; third, by optimizing intestinal morphology and structure, increasing absorptive surface area and enhancing digestive enzyme activity, thus providing strong support for improved growth performance.

Currently, research on the application of CAE in broiler production remains limited. This study compared the effects of CAE with florfenicol exposure in broilers, and the molecular mechanisms underlying the observed phenotypes were not fully dissected. Future studies incorporating pathogen challenge models and pharmacodynamic assessments would deepen our understanding of the biological properties of CAE. Further investigations are also required to identify the key bioactive components of CAE and clarify their respective roles and mechanisms responsible for the beneficial effects.

## 5. Conclusions

This study investigated the effects of CAE in broiler production via a 42-day feeding trial, including a blank control group, a florfenicol group, and three graded CAE treatment groups, with indicators covering growth performance, immunity, antioxidation, intestinal function, and cecal microbiota detected.

Results showed that CAE supplementation enhanced the antioxidant capacity, immune response, and intestinal digestive function of broilers, maintained intestinal mucosal barrier integrity, and altered cecal microbiota composition, accompanied by improved growth performance.

Significant linear and quadratic regression relationships were observed between CAE dosage and multiple indicators, indicating a dose-dependent effect of CAE and the existence of an optimal dosage range for broilers. The 2.5 g/L CAE dose exhibited the best overall effect among the tested groups.

## Figures and Tables

**Table 1:** Composition and nutrient levels of the basal diet (air-dry basis, %).

Item	1 to 21 Days of Age	22 to 42 Days of Age
Corn	58.62	62.74
Soybean meal	34.90	29.88
Soybean oil	2.83	3.58
Limestone	1.48	1.60
Sodium bicarbonate	0.98	1.00
Lys·HCI	0.25	0.27
DL-Met (98%)	0.17	0.16
L-Thr (98%)	0.07	0.07
NaCI	0.30	0.30
Premix ^(1)^	0.40	0.40
Total	100	100
ME/(MJ/kg) ^(2)^	12.28	12.59
CP	21.22	19.12
Ca	0.98	0.94
TP	0.65	0.58
AP	0.42	0.38
Lys	1.18	1.12
Met	0.46	0.42

TP: Total Phosphorus; AP: Available Phosphorus. ^(1)^ The premix provides the following per kilogram of feed: vitamin A, 15,000 IU; vitamin D3, 3000 IU; vitamin E, 100 mg; vitamin K3, 3 mg; vitamin B1, 5 mg; vitamin B2, 7.0 mg; vitamin B6, 5.0 mg; vitamin B12, 0.07 mg; pantothenic acid, 30 mg; nicotinic acid, 45 mg; biotin, 0.5 mg; folic acid, 1 mg; Fe, 80 mg; Cu, 12 mg; I, 0.55 mg; Zn, 105 mg; Mn, 102 mg; Se, 0.3 mg. ^(2)^ Metabolic energy is a calculated value, while the rest are measured values.

**Table 2:** Primer sequences for real-time quantitative PCR.

Gene	Primer Sequence (5′→3′)		GenBank
GAPDH	F:TCCATGCCATCACAGCCACAC	R:ATGACTTTCCCCACAGCCTTAGC	NM_204305
Occludin	F:GCTGAGATGGACAGCATCAA	R:CCTCTGCCACATCCTGGTAT	NM-205128
Claudin-1	F:TTTGGATCAGCCCTCTTCATTGG	R:GCTGGGTGGGTAGGATGTTTC	NM_001013611
ZO-1	F:TGGCAATCAACTTTGGGTAGCA	R:ATCCACAGAGGCAACTGAACCATA	XM_046925214

**Table 3:** Metabolites of CAE characterized by LC-MS/MS.

Name	Formula	Median *m*/*z*	Median Retention Time	Class	Area
Rhusflavone	C_30_H_22_O_10_	543.1321	24.2	Flavonoids	5,063,189,941
(4S,4aR,7aR,8R,9aR)-4,4a,7a,8,9,9a-Hexahydro-4-hydroxy-3-(hydroxymethyl)-4a,8-dimethylazuleno(6,5-b)furan-2,5-dione	C_15_H_18_O_5_	543.1321	147.0	Sesquiterpenoids	7,136,471,688
9-Oxo-10(E),12(E)-octadecadienoic acid	C_18_H_30_O_3_	295.2267	280.9	Fatty Acids and Conjugates	2,704,844,483
Malic acid	C_4_H_6_O_5_	133.0142	25.3	Hydroxy acids	3,779,562,474
(−)-3,5-Dicaffeoyl quinic acid	C_25_H_24_O_12_	515.1202	185.3	Other Phenylpropanoids	3,095,208,623
Butyl ethyl malonate	C_9_H_16_O_4_	187.0978	199.9	Fatty Acids and Conjugates	2,602,290,172
13(S)-HODE	C_18_H_32_O_3_	295.2281	280.4	Eicosanoids	4,127,373,658
(10E,12E)-9-hydroxyoctadeca-10,12-dienoic acid	C_18_H_32_O_3_	295.2281	280.4	Eicosanoids	4,127,373,658
(2E,4Z)-9-oxooctadeca-2,4-dienoic acid	C_18_H_30_O_3_	293.2126	280.6	Fatty Acids and Conjugates	2,886,294,259
Linoleic acid	C_18_H_32_O_2_	279.2332	315.0	Fatty Acids and Conjugates	2,481,446,564

**Table 4:** Effect of CAE on the growth performance of broilers.

Item	Group					SEM	*p*-Value		
	NC	FFC	LCAE	MCAE	HCAE		A	L	Q
1–21 d									
ADG (g/d)	42.41 ^a^	34.54 ^b^	45.18 ^a^	46.68 ^a^	46.58 ^a^	0.8742	<0.01	<0.05	<0.05
ADFI (g/d)	66.09 ^a^	58.83 ^b^	68.08 ^a^	66.81 ^a^	67.88 ^a^	0.9023	<0.01	0.509	0.766
F/G	1.56 ^a^	1.70 ^b^	1.51 ^a^	1.43 ^a^	1.46 ^a^	0.0237	<0.01	<0.05	<0.05
22–42 d									
ADG (g/d)	95.49 ^ab^	90.20 ^b^	98.82 ^a^	99.90 ^a^	96.47 ^ab^	1.0032	<0.05	0.659	0.220
ADFI (g/d)	156.83 ^a^	139.21 ^b^	153.99 ^a^	148.29 ^ab^	148.00 ^ab^	1.8411	<0.05	0.055	0.156
F/G	1.64	1.54	1.56	1.48	1.53	0.0175	0.054	<0.05	<0.05
1–42 d									
ADG (g/d)	68.75 ^b^	61.96 ^c^	71.83 ^ab^	73.35 ^a^	71.47 ^ab^	0.7801	<0.01	0.062	<0.05
ADFI (g/d)	111.46 ^a^	99.02 ^b^	111.03 ^a^	107.55 ^a^	107.94 ^a^	1.1369	<0.01	0.087	0.236
F/G	1.60 ^ab^	1.62 ^a^	1.53 ^bc^	1.46 ^c^	1.50 ^c^	0.0153	<0.01	<0.01	<0.01

^a,b,c^ Means, within each row with different superscripts, are statistically significantly different (*p* < 0.05). The NC group was allowed to freely drink tap water; the FFC group was given 0.15 g/L florfenicol solution in drinking water; the LCAE, MCAE and HCAE groups were respectively given 1.25, 2.5 and 5 g/L CAE in drinking water; SEM, standard error of the mean; A, *p*-value of one-way ANOVA; L, *p*-value of linear analysis; Q, *p*-value of quadratic analysis. (the same below). *n* = 8.

**Table 5:** Effect of CAE on the immune organ indices of broilers (mg/g).

Item	Group					SEM	*p*-Value		
	NC	FFC	LCAE	MCAE	HCAE		A	L	Q
21 d									
spleen index	1.09 ^a^	1.07 ^a^	1.15 ^ab^	1.25 ^b^	1.23 ^b^	0.0176	<0.01	<0.01	<0.01
thymus index	2.84 ^ab^	2.51 ^b^	2.90 ^a^	3.12 ^a^	3.10 ^a^	0.0487	<0.01	<0.05	<0.05
bursa of Fabricius index	1.52 ^b^	1.26 ^c^	1.67 ^ab^	1.76 ^a^	1.79 ^a^	0.0356	<0.01	<0.01	<0.01
42 d									
spleen index	1.01	1.13	1.07	1.03	1.07	0.0329	0.824	0.688	0.923
thymus index	2.82 ^ab^	2.74 ^b^	2.81 ^ab^	3.03 ^a^	3.04 ^a^	0.0393	<0.05	<0.05	<0.05
bursa of Fabricius index	0.58 ^bc^	0.51 ^c^	0.60 ^abc^	0.67 ^ab^	0.69 ^a^	0.0140	<0.01	<0.01	<0.01

^a,b,c^ Means, within each row with different superscripts are statistically significantly different (*p* < 0.05). *n* = 8.

**Table 6:** Effect of CAE on the jejunal histomorphology of broilers.

Item	Group					SEM	*p*-Value		
	NC	FFC	LCAE	MCAE	HCAE		A	L	Q
21 d									
VH (μm)	728.26 ^ab^	639.81 ^c^	721.93 ^b^	765.18 ^a^	769.03 ^a^	9.5794	<0.01	<0.01	<0.01
CD (μm)	152.59 ^ab^	156.88 ^a^	148.86 ^ab^	141.12 ^b^	144.63 ^ab^	1.7067	<0.05	<0.05	0.108
VH/CD	4.78 ^b^	4.08 ^c^	4.86 ^b^	5.44 ^a^	5.32 ^a^	0.0980	<0.01	<0.01	<0.01
42 d									
VH (μm)	1099.06 ^b^	1016.02 ^b^	1225.08 ^a^	1246.59 ^a^	1253.97 ^a^	19.9879	<0.01	<0.01	<0.01
CD (μm)	125.22 ^ab^	112.17 ^c^	128.53 ^a^	124.27 ^ab^	118.80 ^bc^	1.3950	<0.01	0.081	<0.05
VH/CD	8.78 ^c^	9.07 ^c^	9.56 ^bc^	10.03 ^ab^	10.56 ^a^	0.1499	<0.01	<0.01	<0.01

^a,b,c^ Means, within each row with different superscripts are statistically significantly different (*p* < 0.05). *n* = 6.

## Data Availability

The original contributions presented in this study are included in the article. Further inquiries can be directed to the corresponding authors.
